# Direct and Indirect Factors Influencing Cat Outcomes at an Animal Shelter

**DOI:** 10.3389/fvets.2022.766312

**Published:** 2022-06-07

**Authors:** R. J. Kilgour, D. T. T. Flockhart

**Affiliations:** ^1^Urban Wildlife Institute, Lincoln Park Zoo, Chicago, IL, United States; ^2^Department of Animal Sciences, Purdue University, West Lafayette, IN, United States; ^3^Flockhart Consulting, Saskatoon, SK, Canada

**Keywords:** adoption, structural equation modeling, shelter intake, Washington, D.C., intake location

## Abstract

Animal shelters play a vital role for pets, such as transitioning animals between homes, from outdoor communities into homes, caring for unadoptable and community animals, and providing a breadth of veterinary and welfare services. The goal of shelters is to move cats to their appropriate outcome as quickly as possible, which for many animals, is to rehome them as quickly as possible through adoption. Therefore, the ability to identify pre-existing factors, particularly those occurring outside the walls of the shelter, which result in specific outcomes is vital. In this study, we used structural equation modeling to test four hypotheses addressing how to predict cat outcome from a shelter in Washington, D.C. We developed four hypotheses that described how cat outcomes could be predicted, based on four general factors: (1) The characteristics of the cats; (2) The location of origin; (3) The type and date of intake; (4) The length of stay. Using 4 years of data from the Humane Rescue Alliance in Washington, D.C., we found support for each of our hypotheses. Additionally, we tested and found support for a global model, which comprised an amalgamation of our all our predictors. From the global model, we can conclude that many factors are at play in predicting cat outcomes in this shelter and very likely in many others as well. Critically, these factors are interconnected, indicating, for example, that cat characteristics mediate the influence of intake location on outcome type. Furthermore, our study highlights the importance of incorporating influences beyond the shelter when attempting to understand cat outcomes. Therefore, to modify cat outcomes most efficiently, such as increasing adoption probabilities, our results show that efforts may be most effective when incorporating multiple factors.

## Introduction

Animal shelters play a critical role in addressing companion animal welfare by establishing pets with new owners, returning pets to previous owners, identifying and monitoring stray populations, and euthanizing animals in an ethical manner when necessary. In 2019, shelter intake for each animal shelter in the United States averaged more than 1,500 animals, where cats accounted for 49% of intakes (1). In 2016, it was estimated that 25% of households in the US had pet cats, with 31% of those originated from an animal shelter or rescue group (2). In the same year, 7% of households relinquished their cat to an animal shelter (2). Shelters provide new homes for cats, with 61% of cat intakes resulting in adoption (1). Animal shelters may also provide essential services for stray cats in and outside of the shelter. The vast majority of the 48% of stray animal intake in the US are cats (1), Although only a small proportion of stray cats are returned to their owners (only about 5% in 2019, 1), animal shelters may provide the opportunity to improve welfare for stray cats through the provision of targeted trap-neuter-return and return-to-field programs (3–5). It is clear that animal shelters act as a vital transition place for cats, providing numerous roles in the community: adopting relinquished cats to new owners; transitioning cats from stray status to ownership through adoption; supporting stray populations through return to field programs; managing stray cats in the environment *via* trap-neuter-return; and providing humane euthanasia as necessary (6). Additionally, many shelters offer veterinary care services, often providing reduced or no-cost sterilization (2).

Capacity is a major issue in many animal shelters and many shelters are functioning over their capacity to provide adequate care for the animals (7). When shelters function over capacity, this adds stress to the shelter resources, the animals residing at the shelter, as well as to the shelters' staff. Programs such as Capacity for Care focus on modifications to shelter policies and practices and are making major strides in rectifying this issue (8, 9), though challenges persist. Accessing veterinarians with shelter animal knowledge and limited financial support which compromise cat physical and psychological health, making the welfare of animals in shelters of major concern (10). Therefore, identifying ways of most effectively addressing shelter animal outcomes and exploring the importance of factors occurring beyond the specific practices of the shelter are crucial.

A common goal for shelters is to maximize live outcomes, to reduce the length of shelter stays, and to implement programs which assist in achieving these goals. Identifying predictive components which result in different outcomes are vital, and typically, the focus is on characteristics of the cats as well as the type of intake. Regarding the physical attributes of the cat, coat color is considered a strong predictor of adoption (11–13). Age is a prominent factor as well, where younger cats are more likely to be adopted than older cats (14–16). Length of stay is a commonly used metric among shelters, where shorter lengths of stay increase shelter efficiency by increasing animal turnover, improves animal welfare, and reduces risk of illness. While some studies find that coat color influences length of stay (14, 16), others do not (15). Breed (exotic vs. domestic shorthairs) and sex influenced length of stay in a case study conducted by Janke et al. (15), however other studies have found no effect of breed and sex on outcome (16). In addition, the type of intake may also be a strong predictor of outcome type. Clearly, there are many components that can, and often do, influence outcome type, although these effects vary substantially across studies, likely influenced by sample size, geography, and other human-related factors. The inconsistency of results across studies indicate that local conditions are highly relevant, beyond the specifics policies and practices of the individual shelters. It is also likely that these factors work in concert with other predictors, highlighting the need for multivariate models which incorporate the complex interactions between predictors in describing the whole system.

**Figure 1:**
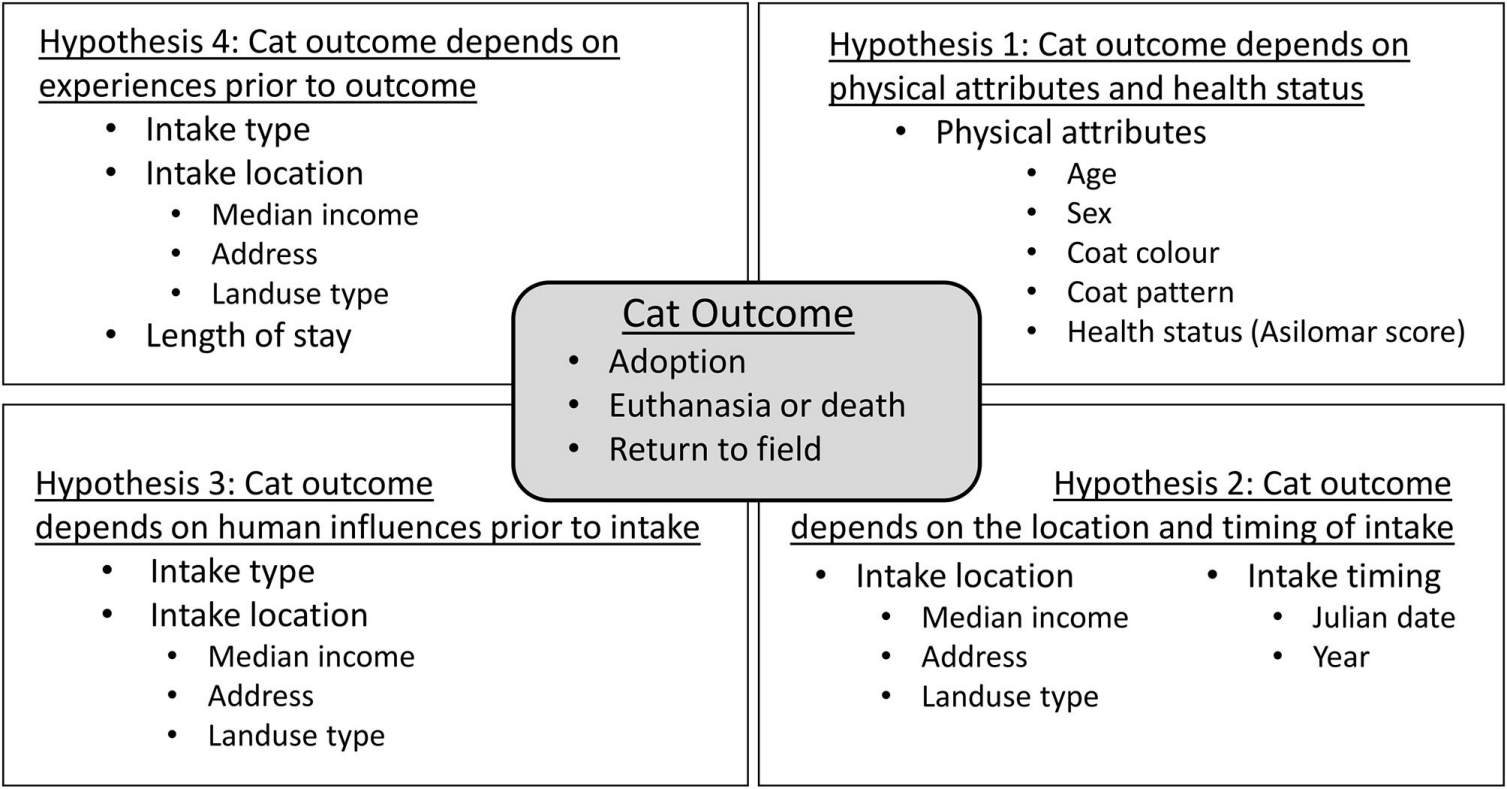
Summary of hypotheses describing the factors used to predict cat outcomes.

Understanding potential factors influencing the outcome of cats in animal shelters is both necessary and challenging, particularly because of the many overlapping factors involved. Previous studies have focused on the use of correlations (17) or linear regression models (especially logistic regression), which predict an outcome based on suites of measured variables (14–16). These studies have substantially contributed to animal welfare research and practice. However, in questions such as ours, the relationships among the measured co-variates are as important as their relationship to the outcome variable of interest, using a whole systems approach. Linear models are unable to capture the complex relationships among multiple explanatory variables, some of which are multifaceted on their own (18, 19). Suitable analytical alternatives to address these data include ensemble methods which include boosted regression trees (20) and structural equation modeling [SEM; (18, 19)] Incorporating SEM in animal welfare is a novel approach in the field and enables the identification of factors related to outcome types as well as the connections and relationships between factors. The strengths of our approach of SEM lie in its ability to use existing knowledge of complex systems to build path models identifying those relationships based on a priori hypotheses. SEM has been previously used to analyze opinions of stakeholders on free roaming cat management techniques (21), but not to animal shelter data. In this study, we applied SEM to test 4 hypotheses describing outcomes of cats in one animal shelter organization. In each hypothesis, we measured both the direct effects of predictor variables on cat outcome as well as the indirect effects between predictors and their resulting effect on outcome. We constructed the following 4 hypothetical models to explain what factors influence cat outcome (Figure 1): (1) Cat outcome depends on the physical characteristics and health status of the cat; (2) Cat outcome depends on the location and timing of intake; (3) Cat outcome depends on human influences (including intake type and intake location characteristics) prior to intake; (4) Cat outcome depends on experiences prior to outcome. Additionally, we constructed a global theoretical model, which represents a combination of all our hypotheses. We used an extensive dataset from a shelter serving the entire Washington, D.C. area, understanding that our model results apply specifically to these data and are not necessarily representative of all shelters. In testing our hypotheses using data from a single location, our goals were to explore the complexity of the relationships between factors influencing outcome, particularly those outside of the shelter. Therefore, we do not address the internal policies of the shelter in this study, as we are interested in examining predictors outside of the shelter itself.

## Materials and Methods

We established four hypotheses to isolate predictors of cat outcomes and how they interact with each other. We followed a two-step process in hypothesis development: first, we assessed leading hypotheses from published literature; second, we discussed each hypothesis with specialists from the field of animal welfare and animal shelters. Finally, we convened a group of workers from the animal shelter where the data was collected to propose, refine, and articulate how each hypothesis may operate in a local context and detail the paths among variables in each model. We used 4 years of data (July 2016 through May 2020) from an animal shelter in Washington, D.C. to test each of our hypotheses.

### Models and Hypotheses

The 4 developed hypotheses offered alternative, though not mutually exclusive, predictions of outcome type (Figure 1). Each hypothesis highlighted the relationships between multiple variables and how they ultimately related to outcome type. We used the following variable categories in constructing our hypotheses, which are described in further detail below: physical attributes and health of the cat; the location of origin (hereafter called intake location); time of intake (date and year); the type of intake; the length of stay at the shelter; and the type of outcome. For each hypothesis, we constructed a theoretical path model that identified the relationships and the direction of the effects between variables (see more details below). Our last model was not a unique hypothesis but was synonymous with a global model, effectively acting as a merger of hypotheses 1–4.

#### Hypothesis 1: Outcome Depends on the Physical Attributes and Health Status

We began with a simple model wherein a cat's outcome is entirely predicted by its physical characteristics and health status at intake (Figure 1). We began with a conceptual variable (also called a latent variable, see below) called physical attributes. This variable represented several physical characteristics of each cat: age, sex, primary coat color, coat pattern of the individual, and the animal's health status at intake, which was judged using the Asilomar score.

#### Hypothesis 2: Outcome Depends on the Location and Date of Intake

In this hypothesis, we assessed how two factors influence outcome: the temporal and the geographic features of intake (Figure 1). We constructed another conceptual (latent) variable called intake location with four components: the latitudinal and longitudinal coordinates of the precise location of origin; the median income in the year of intake for that location; a categorization of the land use type of that location. To account for the temporal components, we incorporated the day of the year, using the Julian calendar, and the year of intake. Based on a preliminary examination of the data and a priori conversations with specialists, we did not hypothesize that these two factors (location and time of intake) would influence the other. Instead, that both these factors influenced cat outcome separately.

#### Hypothesis 3: Outcome Depends on Human Influences Prior to Intake

To address the role that associated humans, including degree of ownership, might influence outcome, we developed a model incorporating intake location and intake type (Figure 1). As in hypothesis 2, we incorporated the location of origin conceptual variable, as this provided some information on the characteristics of the people surrounding the cat. Intake type provided information into the ownership-type (owned or unowned), and thus general degree and type of human interactions. We hypothesized that intake location and intake type would both directly influence outcome type. Additionally, we expected intake location to influence intake type, as geographic characteristics could influence the likelihood of ownership. For example, cats from high density urban areas may be more or less likely to be relinquished by their owner, or cats who arrived as strays may be more likely to have originated from a low-income neighborhood.

#### Hypothesis 4: Outcome Depends on Experiences Prior to Outcome

This hypothesis described how aspects of a cat's life prior to its intake at the shelter as well as its shelter experience influenced its outcome (Figure 1). Pre-shelter life is described through the conceptual variable intake location as well as intake type, as in hypothesis 3. These variables described the degree of ownership, types of human interactions, as well as some information of the owners and the cat's geographic origin. In this hypothesis, we added length of stay, which provided information on shelter experience. We predicted that each factor (intake type, intake location, and length of stay) would directly influence outcome type. Additionally, we expected cats of different intake types to be more likely to have a certain length of stay, for example, stray cats may have longer length of stay, regardless of outcome. Similarly, intake location may have influenced length of stay if certain geographic qualities, such as median income, altered the likelihood of a longer or shorter length of stay. Also, as with hypothesis 3, we expected intake type to be directly influenced by intake location.

#### Global Model

Out last hypothesis described a global model, which allowed us to identify relationships among factors which were not depicted in our hypotheses. Therefore, this model included outcome type and all five of our previously described factors (intake type, physical attributes, intake location, timing of intake, and length of stay). To manage model complexity, we merged intake timing and type into a composite variable called intake attributes, which was described by intake type, intake date, and intake year. In addition to each variable directly influencing outcome type, we further expected length of stay to be influenced by intake attributes, intake location and physical attributes. Intake attributes would be influenced by intake location and, additionally, physical attributes of the cat. The physical attributes describe how a cat's age, sex and appearance influence the type of intake, such as if older cats are more likely to be brought in as owner relinquishment. We also predicted the physical characteristics of a cat to be influenced by the intake location, for example if cats from certain geographic areas are more likely to be of greater or poorer body condition. Cat physical attributes were also expected to be predicted by intake attributes, as stray cats are more likely to be younger and in poorer body condition. This model does not describe any specific hypothesis but demonstrates the interrelatedness of the factors we expected to influence outcome in our previous hypotheses and identifies potential mediating factors.

### Data

To understand predictors of cat outcomes, we used information collected by the Humane Rescue Alliance, Washington, D.C. (HRA) between July 2016 and May 2020 from PetPoint software. The Humane Rescue Alliance is the sole animal welfare and animal control organization serving Washington D.C. and as such the data is assumed to represent a full census of relevant individual cats with no requirement for sampling to draw conclusions across Washington D.C. The shelter will intake cats for a variety of reasons including relinquishment by owners, acquisition or presentation of lost, stray, or abandoned animals, temporary intake for TNR surgery, or animal control seizure. Outcomes may include return to field, return to owner, adoption to a new owner, or euthanasia for medical or safety reasons. Data used in the current study included animal identification number, sex, date of birth, primary color, intake date, intake type, intake location, outcome type, outcome date and were provided by HRA records. When a cat had been brought to HRA multiple times, we included only its first record in our analysis. The eligibility criteria described in each category below resulted in the removal of 4,937 entries from the initial PetPoint dataset. A summary of the data, corresponding to outcome types, can be found in Table 1.

**Table 1:** Summary statistics for outcome types across variables used in this study, from July 2016 through May 2020.

**Variable**	**n**	Outcome: adopted^**^(*n* = 8,445; 75.9%)	**Outcome: Died (*n* = 1,736; 15.6%)**	**Outcome: return to field (*n* = 945; 8.5%)**
Age (years) (mean ± standard error)	11,126	2.0 ± 0.03	7.0 ± 0.2	2.3 ± 0.1
**Intake Type**
^**^Owner/guardian surrender	3,969	2,844 (71.7%)	1,004 (25.3%)	121 (3.05%)
Return	52	46 (88.5%)	5 (9.6%)	1 (1.9%)
Seized/Custody	180	166 (92.2%)	8 (3.3%)	6 (4.4%)
Stray	6,925	5,389 (77.8%)	719 (10.4%)	817 (11.8%)
**Primary Color**
Black	3,723	2,810 (75.5%)	601 (16.1%)	312 (8.4%)
Brown	2,283	1,764 (77.3%)	305 (13.4%)	214 (9.4%)
Buff	282	233 (82.6%)	33 (11.7%)	16 (5.7%)
Cream	141	110 (78.0%)	15 (10.6%)	16 (11.3%)
Grey	2,389	1,778 (74.4%)	407 (17.0%)	213 (8.9%)
Orange	1,118	831 (74.3%)	192 (17.2%)	95 (8.5%)
White	1,087	868 (79.9%)	148 (13.6%)	71 (6.5%)
^**^Other	94	51 (54.3%)	35 (37.2%)	8 (8.5%)
**Sex**
^**^Female	5,487	4,264 (77.7%)	802 (14.6%)	421 (7.7%)
Male	5,173	4,173 (80.7%)	815 (15.7%)	443 (8.6%)
Unassigned	208	8 (3.8%)	119 (57.2%)	81 (38.9%)
**Coat Pattern**
Calico	317	230 (72.5%)	60 (18.9%)	27 (8.5%)
Dilute	260	222 (85.4%)	27 (10.4%)	11 (4.2%)
Marble	141	110 (78.0%)	20 (14.2%)	11 (7.8%)
Point	146	120 (82.2%)	11 (7.5%)	15 (10.3%)
Solid	382	301 (78.8%)	49 (12.8%)	32 (8.4%)
Tabby	3,708	2,946 (79.4%)	448 (12.1%)	314 (8.5%)
Tiger	142	89 (62.7%)	38 (26.8%)	15 (10.6%)
Torbie	301	261 (86.7%)	22 (7.3%)	18 (6.0%)
Tortoiseshell	446	352 (78.9%)	61 (13.7%)	33 (7.4%)
Tuxedo	478	360 (73.6%)	65 (13.6%)	53 (11.1%)
Van	151	138 (91.4%)	10 (6.6%)	3 (1.9%)
^**^Other	125	95 (76.0%)	14 (11.2%)	16 (12.8%)
N/A	4,529	3,221 (71.1%)	911 (20.1%)	397 (8.8%)
**Asilomar status**
^**^Healthy	1,793	1,570 (87.6%)	78 (4.3%)	145 (8.1%)
Treatable-manageable	52	30 (57.7%)	7 (13.5%)	15 (28.8%)
Treatable-rehabilitatable	151	98 (64.9%)	23 (15.2%)	30 (19.9%)
Unassigned	8,760	6,701 (76.5%)	1,309 (14.9%)	750 (8.6%)
Unhealthy-Untreatable	338	20 (52.6%)	317 (93.8%)	1 (0.3%)
N/A	32	26 (81.3%)	2 (6.2%)	4 (12.5%)
Intake date (Julian) (mean ± standard error)	11,126	193.4 ± 1.0	190.7 ± 2.4	188.5 ± 3.3
**Intake Year**
^**^2016	2,245	1,698 (75.6%)	371 (16.5%)	176 (7.8%)
2017	2,801	2,047 (73.1%)	456 (16.3%)	298 (10.6%)
2018	2,763	2,136 (77.3%)	410 (14.8%)	217 (7.8%)
2019	2,729	2,131 (78.1%)	411 (15.1%)	187 (7.9%)
2020	588	434 (73.8%)	87 (14.8%)	67 (11.4%)
Length of stay (mean ± standard error)	11,126	30.0 ± 0.4	6.9 ± 0.5	13.1 ± 0.7
**Landuse Type**
^**^Natural	43	32 (74.4%)	7 (16.3%)	4 (9.3%)
Developed/high intensity	1,643	1,305 (79.4%)	257 (15.6%)	81 (4.9%)
Developed/low intensity	2,902	2,229 (76.8%)	403 (13.9%)	270 (9.3%)
Developed/medium intensity	6,243	4,648 (74.5%)	1,031 (16.5%)	564 (9.0%)
Developed/open space	295	231 (78.3%)	38 (12.9%)	26 (8.8%)
Median Income (at intake) (mean ± standard error)	11,126	57,544.9 ± 368.2	65,391.5 ± 938.5	57,025.7 ± 1,020.3

*For continuous variables, mean and standard error are provided. For categorical variables, counts, and proportions (in percentage) are described*.

** * Indicates reference category*.

#### Outcome Type

We considered three possible outcomes for cats in our study: Adoption, Died and Return-to-field. Outcomes were merged into these three categories to represent the major categories of outcome, reflecting live outcomes, death, and potential degree of ownership as well as to account for some outcome categories have small sample sizes relative to the major outcome types. Live outcomes were classified based on the degree of ownership and human responsibility. Adoption includes cats who were adopted into new homes and cats who were returned to previous owners. Died includes individuals who were euthanized and individuals who died by natural causes after intake. We did not include individuals who were classified as dead on arrival, as their outcome was predetermined on arrival. Return-to-field describes cats who were brought to HRA as strays and were subsequently returned to their outdoor location. While return-to-field is a live outcome, the cats with this outcome generally were not adoptable and therefore have a lower potential for ownership compared to those cats that were adopted. We only included cats whose live outcomes were in the Washington, D.C. area. The most common outcome was adoption, followed by those who died and lastly return to field (*n* = 8,445; 1,737; and 945, respectively, Table 1; Supplementary Figure 1). Outcome type was our variable of interest in each model, and as we were most interested in adoption, we used this as our reference category in our models.

#### Physical Attributes

Consistent with previous studies, we predicted that various characteristics of the cat itself influenced the type of outcome for that individual. As such, we included data on age at time of outcome, sex, two features of the cat's appearance (the coat pattern and primary coat color) and the health status at intake, recorded as the Asilomar score. Age was estimated based on dental development, dental health (such as gum wear and plaque buildup), and tooth wear, techniques commonly used to estimate cat age (22). We included only cats under the age of 30. Asilomar scores refers to the standardized 4-point scale describing the health status of the cat: healthy, treatable-rehabilitatable, treatable-manageable, and unhealthy/untreatable and have been used in other studies to identify health status (23). HRA defined Asilomar scores as based on previously described definitions (24). Asilomar scores could also be classified in “unassigned” if they were not assigned a score immediately upon intake. While specific breeds were not incorporated in this model, the specificity of some coat colors provides some indicator of breed type. Coat length was not incorporated in the physical description. The classification of all components physical attributes was designated by HRA staff at time of intake.

We found a diversity of all attributes among cats included in our analysis (Table 1, Supplementary Figure 2). The average age of cats at intake was 6.0 years (median: 4.9 years). There were nearly equal proportions of male and female cats at intake (female: 49.3%; male: 48.8%; unknown sex: 2.0%). The most common primary coat color was black (33.5% of intakes) and the most common coat pattern was tabby (33.3%). All colors or patterns with fewer than 100 counts were merged and are listed as “Other.” Asilomar score at intake was predominantly Healthy (16.1%), although most cats were listed as “unassigned” (78.7%).

#### Intake Date

We hypothesized that the date and year of intake influenced the outcome of a cat. For this analysis, we separated date into the year of intake and the Julian calendar day of the year, a scale ranging from 1–365, where 1 is January 1 and 365 is December 31. Using these two variables we were able to capture differences between years and days within years (and thus seasonality). Year of intake was between 2016 and 2020, any cats whose intake year was prior to our date range were removed from this analysis. There were 6 months of intake data for 2016, 5 months for 2020, and 12 months for 2017, 2018, and 2019. It should further be noted that there was a sharp decline in intake numbers in 2020 as a result of the SARS-CoV-2 pandemic. With respect to Julian calendar date, most intake dates occurred in spring, summer, and early fall, with pulses of intakes in late spring (May/June; days 120–180, approximately) and again in early autumn (September; days 240–273, approximately) (Table 1; Supplementary Figure 3).

#### Location of Origin

Upon intake, information the address of origin for each cat is collected. Intake address refers to the home address of the owner if intake type was owner relinquish, or the address, or nearest intersection, where the cat was found. We included only cats who originated from within the boundaries of Washington, D.C, and whose precise intake location was noted. A map of intake locations can be found in Supplementary Figure 4.

In our analyses, we included the precise geographic location, as described through latitude and longitude coordinates, as well as median income and landuse type. From the street address, we could obtain other geographic and socioeconomic information, which were described based on a 400 m-by-400 m grid. For each square, we summarized the land use, sum of residential units and the annual median household income. Median income and the number of households in each grid square was determined based on the year of cat intake using the census tract-level 2016–2019 American Community Survey (ACS) 5-Year Estimates from the U.S. Census Bureau (25). Income data for 2020 was from an ESRI data product using the same methodology as the ACS data (26). Landuse type was obtained from the National Land Cover Database (27) and reclassified into 5 levels: high-intensity developed; medium-intensity developed; low-intensity developed; open-space developed; and natural, which described areas with forest, croplands or waterways (Table 1; Supplementary Figure 5). Median income ranged from $13,595–$242,208 (median: $46,594; mean: $57,727) and most cats originated in medium intensity developed landuse types (56.1%) (Table 1; Supplementary Figure 6).

#### Type of Intake

We expected a strong relationship between intake type and outcome type, both directly and indirectly. Intake type refers to the classification of the reason for which the cat is brought to the shelter. Intake categories in our study were as follows: owner surrender, seized/custody; return (recently adopted cats who are being returned to the shelter within 30 days following adoption), and stray (cats with no known owner). The majority of intake types were stray (61.0%), and owner relinquish (36.9%; Table 1; Supplementary Figure 7).

#### Shelter Attributes: Length of Stay

Following the date of intake, a cat's outcome is likely influenced by elements of its shelter stay. In our study, we incorporated the length of stay as a predictor of outcome type. For cats in our study, the average length of stay was 24.9 days (range: 0–861 days; median: 12.0 days; Supplementary Figure 8), however this varied across outcome types (Table 1).

### Structural Equation Models

Our data were a combination of continuous and categorical, which comprised both endogenous variables (which are described by other variables and have arrows directed toward them) and exogenous variables (which are used to describe other variables, and only have arrows directed away them). All continuous variables (age, median income, Julian calendar dates) were converted to z-scores to reduce homoscedasticity in latent regression models. Categorical variables were either binomial or incorporated as dummy variables.

We used a combination of observed, latent, and composite variables in our models. Observed variables refer to variables which have been directly measured. Latent variables (previously called conceptual variables) refer to variables which are not directly observed but instead are inferred or estimated through other observed variables. Latent variables are particularly useful when describing a concept which cannot be objectively quantified and observed variables capture only a portion of the variance. In other words, latent variables capture broad concepts, where some portion of the concept is unmeasured or unmeasurable. We used latent variables in our models to describe the physical attributes of individuals as well as their locations of origin. Therefore, we expected that there may have been other variables describing the physical attributes and the intake location which were important in the model but unmeasured and thus not included here (19). In the construction of our latent variables, we did not include covariances between the observed variables. We had no expectation to account for this variance *a priori*. Composite variables are construct variables that are described in their entirety by a collection of observed variables, and thus have no variance. In contrast to latent variables, composite variables do not described concepts but act as a collection of related variables which can manage model complexity and aid in generalizing variables (19).

We used diagonally weighted least squares (DWLS) to estimate model parameters, as suggested for use among ordinal variables (28). In all models, we did not impose restrictions on y-thresholds. Model fit was assessed using three criteria: Comparative Fit Index (CFI), Root Mean Square Error of Approximation (RMSEA), and Standardized Root Mean Square Residual (SRMR). CFI indices were considered acceptable at >0.95; RMSEA values were considered acceptable at <0.08 (and <0.05 are considered good); SRMR <0.10 were considered acceptable (29). CFI, RMSEA and SRMR are most appropriate measures of fit for large sample sizes (19). We incorporated robust test statistics that were adjusted for mean and variance using the Satterthwaite approximation, also recommended for categorical variables and large sample sizes (30, 31). Satterthwaite approximations provide robust test statistics, which are reported here, unless otherwise indicated. Standardized parameter estimates and standard errors were estimated through bootstrapping, with 1,000 draws.

Model analysis began with complete models and was subsequently modified to improve fit while keeping within the original hypothesis. Variables were removed if their inclusion prevented estimation of the model. Prior to inclusion in the SEM models, we used confirmatory factor analysis to assess the fit of latent variables, again using CFI, RMSEA and SRMR fit indicators. CFA and SEM were conducted in the lavaan package (32) in R (33).

## Results

Data from cat intakes and outcomes (*n* = 11,126 cats) at the HRA were fit to the proposed models. Confirmatory factor analysis showed both measurement models (physical attributes and intake location) had good model fit to the data (Table 2) and were thus included in structural equation models. As such, we did not account for covariances between observed variables describing each latent variable. Sex was not included in the physical attributes model (Hypothesis 1), as there was insufficient variation in outcomes between male and female cats. That is, there was not a considerable difference in the outcome types between males and females at the HRA (Table 1) Primary coat color at this shelter was not a statistically significant contributor to the physical attributes but was kept in the latent variable as it contributed to model fit. All other observed variables were statistically significant contributors to the latent variables. All observed variables describing intake location had a statistically significant influence, although median income at year of intake was the strongest contributor.

**Table 2:** Summary of model fit criteria for latent variables (cat characteristics and intake location) and structural equation models.

**Model**	**CFI (acceptable >0.90)**	**RMSEA (acceptable <0.05)**	**SRMR (acceptable <0.10)**
Physical attributes	1.00	0.00	0.00
Intake location	0.99	0.04	0.01
Model 1	0.99	0.02	0.01
Model 2	0.99	0.02	0.02
Model 3	0.98	0.04	0.03
Model 4	0.99	0.03	0.02
Model 5 (global)	0.96	0.03^*^	0.03

*Models 1 through 4 test our specific hypotheses and model 5 refers to the global model (see text for details)*.

* * Indicates standard value. All other values represent robust estimates*.

*Post hoc* adjustments were made if initial models were not a good fit, and revised models were nested within original models and were supported by expert opinion and the current literature. Each of our models showed acceptable goodness of fit metrics in all three measures (CFI, RMSEA, and SRMR) (Table 2). Based on how our outcome variable was coded, variables which had negative direct effects on outcome, represented by negative β values in the text, indicate a positive relationship between the variable and the likelihood of adoption. Therefore, negative direct effects refer to increased adoptions at HRA. Likewise, variables which have a positive direct effect on outcome indicate a negative relationship with the likelihood of adoption, or positive direct effects refer to decreased adoptions at HRA. Error terms describe the amount of variance unique to that variable and are incorporated into the calculation of the standardized parameter estimates.

In hypothesis 1, we found that cat physical attributes were significant predictors of cat outcome (β = 0.47, *SE* = 0.02, *p* < 0.01; Supplementary Figure 9). Within the latent variable, age and Asilomar score were the strongest contributors to the effect [age: β = 0.57, *SE* = 0.02, *p* < 0.01; Asilomar score: β = 0.57, *SE* = 0.02, *p* < 0.01)], followed by coat pattern (β = −0.10, *SE* = 0.01, *p* < 0.01). Primary coat pattern had weak and non-significant influences (β = 0.03, *SE* = 0.02, *p* = 0.08) on our latent variable.

In our second hypothesis, examining the temporal and geographic attributes of intake, location of origin was the strongest predictor of outcome type (β = −0.06, *SE* = 0.01, *p* < 0.01; Supplementary Figure 10). Intake year also had a significant effect on outcome type (β = −0.03, *SE* = 0.01, *p* = 0.02), and significantly covaried with Julian date of intake (β = −0.29, *SE* = 0.01, *p* < 0.01). Additionally, Julian date of intake was also a direct predictor of outcome type (β = −0.03, *SE* = 0.01, *p* = 0.02). Within the latent variable, we found all our measured variables had a significant effect, with median income as the strongest contributor (β = −0.84, *SE* = 0.01, *p* < 0.01), followed by landuse type (β = 0.06, *SE* = 0.01, *p* < 0.01). The latitude and longitude coordinates were also statistically significant (longitude: β = −0.02, *SE* < 0.01, *p* < 0.01; latitude: β = −0.02, *SE* < 0.01, *p* < 0.01).

Hypothesis 3 examined the influence of intake location and type on outcome to identify the human component of predicting cat outcome. We found that intake type did not have a significant direct effect on outcome type (β = −0.01, *SE* = 0.02, *p* = 0.67) and that intake location did have a significant and direct effect on outcome (β = −0.06, *SE* = 0.01, *p* < 0.01). Furthermore, intake location had significant influence on intake type (β = 0.05, *SE* = 0.01, *p* < 0.01). The estimates for the measured variables describing location of origin were consistent with the results to hypothesis 2, where all variables were statistically significant and median income was the strongest contributor, followed by land use type (Supplementary Figure 11).

The fourth model for the shelter and pre-shelter experience hypothesis incorporated intake type and length of stay as observed variables. The addition of length of stay altered the relationship between intake type and outcome. Indeed, all our hypothesized connections were statistically significant, although length of stay had the strongest effect on outcome type (β = −0.54, *SE* = 0.03, *p* < 0.01), followed by intake type (β = 0.04, *SE* = 0.01, *p* = 0.01) and intake location (β = −0.03, *SE* = 0.01, *p* = 0.01). As in hypothesis 3, intake location influenced intake type (β = 0.05, *SE* = 0.01, *p* < 0.01) and length of stay was also significantly predicted by both intake type (β = 0.09, *SE* = 0.01, *p* < 0.01) and intake location (β = 0.05, *SE* = 0.01, *p* < 0.01). The intake location latent variable was similarly described by measured variables as in hypotheses 2 and 3 (Supplementary Figure 12).

**Figure 2:**
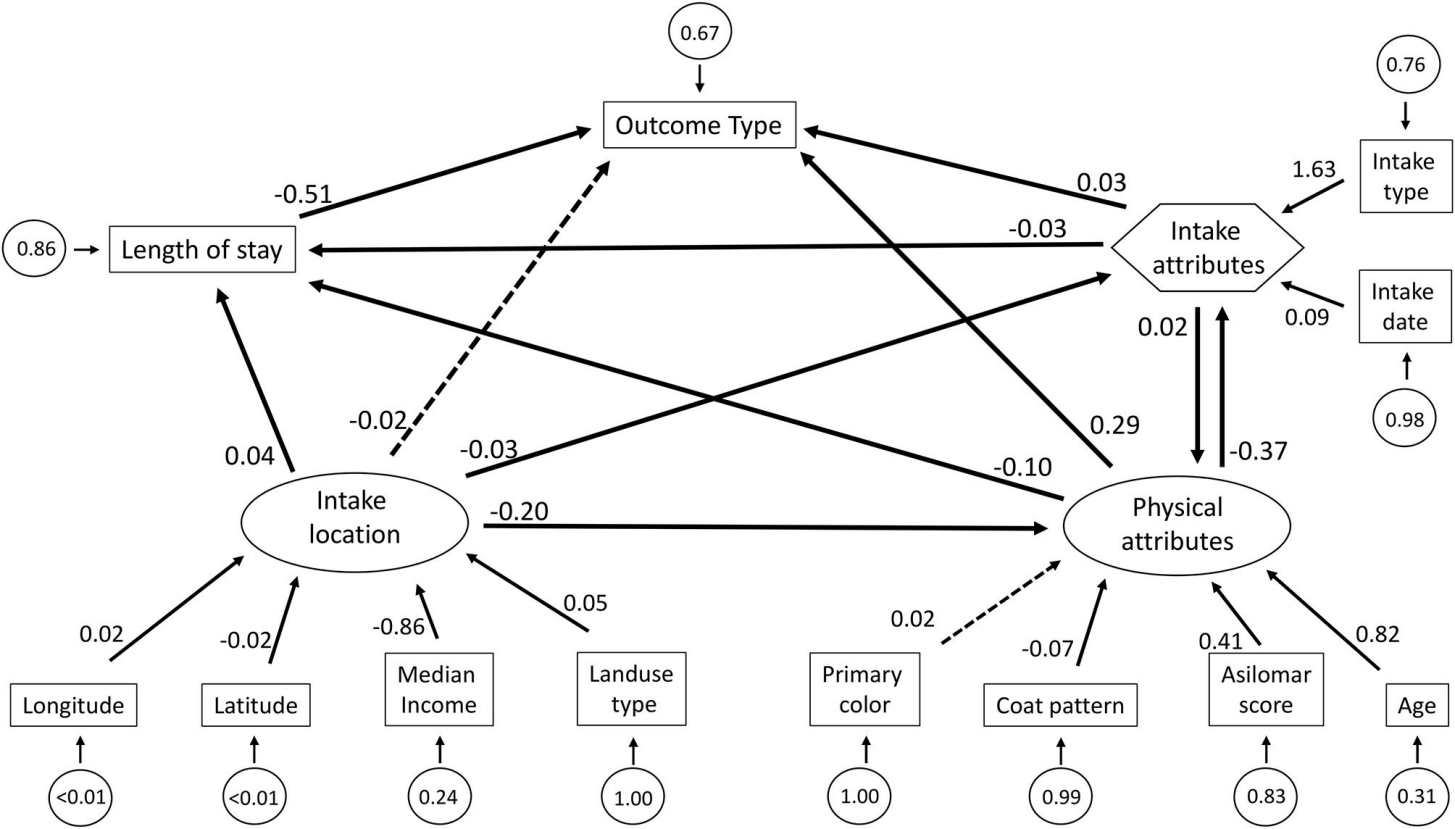
The structural equation model describing our global model predicting cat outcomes. Arrows describe the direction of effect. Solid black arrows are statistically significant at α = 0.05, dotted arrows indicate a lack of statistical significance. Numbers alongside the arrows are standardized path coefficients (beta coefficients). Measured variables are depicted in rectangles, latent variables are depicted in ovals, and composite variables are depicted in hexagons. The error terms, which describe the variance of the associated term, are in circles. Possible outcome types are adoption, death/euthanasia, and return to field. Based on how they were coded, negative coefficients indicate an increased likelihood of adoption and positive coefficients indicate a decrease in likelihood of adoption.

Lastly, we fit our data to a global model, which represented a culmination of our 4 hypotheses (Figure 2). In this model, we incorporated a new composite variable, labeled intake attributes, described by the measured variables intake type and intake date. Cat physical attributes and intake location were incorporated as latent variables, as in previous models. Therefore, we had four variables directly influencing outcome type: intake attributes, length of stay, physical attributes, and intake location. Length of stay had the strongest effect on cat outcome (β = −0.51, *SE* = 0.03, *p* < 0.01). The negative beta value between length of stay and outcome indicates that cats who were adopted tended to have longer lengths of stay than cats with other outcomes (death or return to field), as described in Table 1. We also found a significant effect of physical attributes (β = 0.29, *SE* = 0.01, *p* < 0.01). Intake attributes had a smaller, but still statistically significant influence (β = 0.03, *SE* = 0.01, *p* < 0.01). Our global model demonstrated no significant direct effect of intake location on cat outcome (β = 0.02, *SE* = 0.01, *p* = 0.22). Length of stay was significantly influenced by physical attributes (β = −0.10, *SE* = 0.01, *p* < 0.01), intake location (β = 0.04, *SE* = 0.01, *p* < 0.01), and intake attributes (β = −0.03, *SE* = 0.01, *p* < 0.01). We also found that physical attributes were significantly predicted by intake location (β = −0.20, *SE* = 0.02, *p* < 0.01) and by intake attributes (β = 0.02, *SE* = 0.01, *p* < 0.01). Furthermore, intake attributes were significantly, and strongly, influenced by physical attributes (β = −0.38, *SE* = 0.01, *p* < 0.01) and by intake location (β = −0.03, *SE* = 0.01, *p* = 0.08).

Based on all three model fit statistics we found nearly equal support for all our hypotheses (Figure 1). Therefore, we cannot reject any hypothesis in addressing factors that influence cat outcomes. Additionally, results from the separate hypothesis models were generally reflected similarly in the global model with additional connections among variables demonstrated how the hypotheses interrelate. However, the general consistency of results supports use of the global model as our final model for interpretation and understanding of the complex system, where specific interpretations apply only to the HRA shelter in Washington, D.C.

## Discussion

Animal shelters are often under pressure to maintain efficiency, given constraints on capacity, welfare, and resources. Generally, the ideal outcome for socialized cats coming into a shelter is adoption, although many cats face alternative outcomes, such as being returned to their outdoor location, as with stray cats, and euthanasia or death, with cats who are ill, injured. Therefore, expanding our knowledge of predictors of animal outcomes could greatly improve shelter efficiencies. In this study, we applied 4 years of shelter cat data from one organization to test four different, though not mutually exclusive, hypotheses: first, cat outcome depends on the physical attributes and health status of the cat; secondly, outcome depends on the location and timing of intake; thirdly, outcome depends on human influences prior to intake; fourthly, outcome depends on experiences prior to outcome. Additionally, we tested a global model, which was an amalgamation of our hypotheses, to examine the interactions between our predictors. We used structural equation modeling to explore each hypothesis, based on five general factors: cat characteristics (age, body condition, coat color, and coat pattern), location of origin (latitude, longitude, median income and landuse type), intake information (type and date), and length of stay. We found that each of our four hypotheses were supported by our models, indicating that factors represented in each model were important in predicting cat outcomes. Given that all our hypotheses were supported by our models, and that the hypotheses were not mutually exclusive, we concluded that the global model provides the best description of the system. While the results to our models are specific to the factors at a specific shelter (the Humane Rescue Alliance) in a specific location (Washington, D.C.), we provide here an overarching understanding of how cat specific factors outside of the shelter interact to predict cat outcome, both directly and indirectly. We specifically do not include policies and practices internal to the shelter in our analysis. Our goal was to demonstrate both the importance of factors beyond the animal shelter in predicting outcome, as well as to encourage a similar exploration of factors at other shelters and locations.

Using SEM allows us to identify and quantify relationships within a whole system and provides novel insights in understanding outcome type. Figure 2 highlights the complexities and nuances involved in predicting cat outcomes, demonstrating the importance of mediator and indirect effects in describing the whole system. Our global model results demonstrate two key points: first, we find that there is no singly important factor. That is, all five factors that we considered provided a significant contribution to the system, even if not directly influencing outcome. Secondly, the interconnectedness of the global model demonstrates how efforts to enact change in one part of the system can result in changes elsewhere, emphasizing that there are many ways to change outcome probabilities. Unlike most other studies predicting shelter outcomes which have relied on predictors specific to the animals or the shelter (34), our approach incorporates factors outside the shelter and animal which may be important. While this older approach is logical, the results can only inform modifications to practices within the shelter (35).

Consistent with other studies, we found evidence that in Washington, D.C., intake type, potentially indicating degree of ownership, and physical attributes of cats are both important components of the system relating to outcomes. We also found that these two factors interact in how they influence outcome. Marston & Bennett (36) described the relationship between cat characteristics, intake type and cat outcome, determining that stray cats who are adults and in poor body condition are more often euthanized. We further found that these two factors also play an important role in influencing length of stay in D.C. Additionally, we found a strong and significant relationship between length of stay and cat outcome, where cats with longer lengths of stay were more likely to be adopted. It is worth noting the substantial variation in lengths of stay across outcome types at HRA, as described in Table 1. Lengths of stay can be determined by multiple factors including policies specific to individual shelters and we used length of stay as a proxy. For example, many shelters have specific hold times for certain outcomes, such as if stray cats are brought to a shelter for TNR before being returned to their original location. Shelters may also impose a hold period prior to euthanasia. We did not incorporate shelter policies and specific practices in our model, as variation in practices between shelters highlight the challenges in identifying patterns generalizable to all shelters. Our global model described how length of stay is influenced by myriad factors, most prominently by cat characteristics (Figure 2). Older cats brought to shelters as a result of owner relinquishment often have a longer duration of stay or are less likely to be adopted (37). Our global model indicates that in our system, age effects on adoption likely are influenced by the location of origin of the animal.

Intake location had an important role in our global model, although it did not have a direct effect on outcome type once all five factors were combined. GIS (Global Information System) data has been used to explore variation in cat abundance across urban areas (38, 39) where interpretation is often based on the landuse type. Spencer et al. (40) used GIS data to explore demographic information regarding the location of origin of stray dogs and cats and attribute local densities of abundance across Alachua County, Florida. Isolating the location of origin of cats upon intake can be used to determine where shelter resources may be most efficient. Consistent with previous findings, our model indicates that in Washington, D.C., the intake location does predict intake type, supporting the notion that strays or relinquished cats typically arrive from specific areas. However, our model also indicates there was no direct relationship between location of origin and outcome. That is, for example, stray cats may more commonly originate in certain geographic areas, we found no evidence that those stray intakes are more likely to result in a specific outcome in our system. Instead, we see how the effect of intake location on outcome is mediated by our other variables of interest, most strongly with characteristics of the cat. We also found that intake location predicts length of stay as well as intake characteristics. It is therefore clear that intake location is an essential component in predicting outcomes in this system, but efforts to change likelihood of adoption must incorporate other components as well. This relationship is described in previous studies describing how financial constraints influence decisions to relinquish cats to shelters (41). Our model results indicate that median income is the strongest contributor to the intake location latent variable, followed by landuse type. Additionally, it should be noted that our geographic locations were very precisely reported based on lat/long coordinates, as this allows us to capture the considerable geographic variation found in Washington, D.C. Given the variation in wealth across the Washington, D.C. area, this may not reflect other communities across the United States. However, a cat's intake location is rarely included in studies on determinants of cat outcome, and we encourage other studies to incorporate this factor when possible.

The limited resources of animal shelters and the ongoing demand for their services, require shelters to develop optimal strategies for allocating resources. Many shelters are often overburdened, such as in areas with increasing stray cat populations [for example, (7)]. As a result of the high volume of cats in need of shelter services and the limited resources of the shelter, the concept of optimality has been a major focus in the last several years. The Capacity for Care (or, C4C) management model, based on the guidelines established by the Association of Shelter Veterinarians (8), emphasizes the use of optimal strategies within the shelter to increase the volume of cats in their care over a set period of time. The goal of C4C is to increase the efficiency of shelters by focusing on reducing lengths of stay, increasing adoptions, and improving health and welfare of shelter cats (9, 42, 43). The suggestions described in C4C are based on changes within the shelter: the physical structure, housing, as well as general management (44, 45). That is, these refer to modifications that can be made within the shelter and through shelter policies and have resulted in improvements to the functionality of many shelters. Although internal policies were not considered in our analysis, there is little doubt that they influence cat outcome. Given that we relied on data from a single shelter, we therefore could not incorporate variation in internal policies in our study. In contrast, our model mostly considered factors outside of the shelter, and efforts such as C4C would be reflected in length of stay and the frequency of outcome types. As a result, our model provides a road map for shelters to understand the system and institute changes in the most effective way. For example, if older cats from low-income areas are more likely to require euthanasia or to have a very long length of stay at the shelter, then initiatives to support geriatric care (or other strategies) targeted to those geographic areas may be the best way of altering outcomes.

While this study provides a novel perspective on how many factors can influence cat outcomes, there were several limitations to our study. It should be noted that our results were derived from the data of a single shelter that serves to entire Washington D.C. area. Using a data from only HRA came with several benefits, such as that we could ensure relative consistency in data collection, and Washington D.C. encompasses a large geographic area, providing substantial variation in our geographic data. However, the results found here may not be applicable to smaller urban centers, or locations with different climactic, demographic, and geographic profiles. While our study area provided extensive variation in geographic factors such as median income and landuse type which are useful for SEM, basing our results on a single location prevents us from considering other factors. For example, climatic and seasonal changes have substantial impacts on stray cat population abundance and reproduction [(46), though see (47)], as well as the relative intensity of urbanization (48). Therefore, we discourage the use of our specific model results for determining allocation of resources in other shelters. Instead, we encourage the use of similar models to understand the complexities of the local systems. In using data from only one shelter, we were also constrained by the modes of data entry occurring here, particularly around how Asilomar score was categorized. Asilomar score was a strong and significant predictor in all models (Supplementary Figure 7; Figure 2) and there was substantial variation in outcome types across the categories (Table 1). However, we recognize the limitations in interpretation of this variable, given the proportion of “unassigned” cats (Supplementary Figure 2E). Other studies have considered how the transfer of animals from shelters to rescue groups increases adoption (49), which was also not considered in our analysis. We summarized shelter experience by a single measured variable, length of stay, which does not encapsulate many components of the shelter itself.

In this study, we provide a novel perspective toward understanding predictors of shelter cat outcomes. Using structural equation modeling on data specific to Washington, D.C., we found that cat characteristics, type and date of intake, and length of stay had direct influences on outcome type at this shelter and in this region. Further, we found that location of origin played an important role as a mediator in influencing outcome type, although did not have a direct influence. From our global model, we have shown the vast complexity of the system in predicting outcomes in shelters, demonstrating not only the direct effects of multiple factors but also how these factors are themselves interrelated. As shelters often face conflicting demands of intake numbers and capacity, the appropriate allocation of resources to increase live outcomes and minimize euthanasia is vital. While our results refer specifically to Washington, D.C., our model results demonstrate the importance of incorporating factors outside of the shelter in addressing changes to outcome type, highlighting how many components can alter cat outcome. Additionally, as factors are themselves interconnected, increasing resources (such as community programming and support services) to specific cat populations are encouraged to explore relationships between factors, and models may identify the how outcome is intake type, physical characteristics, and intake location. Such model results could highlight a regional importance to distribute resources to, for example, stray cats in low-income areas or geriatric cats in high income areas. We encourage the use of path models in other geographic areas and systems as a means of addressing specific needs in other regions and maximizing the contributions of animal shelters to the communities they serve.

## Data Availability Statement

The original contributions presented in the study are included in the article/Supplementary Materials, further inquiries can be directed to the corresponding author/s.

## Author Contributions

RK and DF conceived of the study, developed hypotheses and models, interpreted the data, and contributed to the writing of the manuscript. RK performed the statistical analysis. All authors contributed to the article and approved the submitted version.

## Funding

Major financial support was provided by: PetSmart Charities, American Society for the Prevention of Cruelty to Animals, The Humane Society of the United States, the Humane Rescue Alliance, Winn Feline Foundation, Maddie's Fund, Cat Depot, and B. Von Gontard.

## Conflict of Interest

DF was employeed by Flockhart Consulting. The remaining author declares that the research was conducted in the absence of any commercial or financial relationships that could be construed as a potential conflict of interest.

## Publisher's Note

All claims expressed in this article are solely those of the authors and do not necessarily represent those of their affiliated organizations, or those of the publisher, the editors and the reviewers. Any product that may be evaluated in this article, or claim that may be made by its manufacturer, is not guaranteed or endorsed by the publisher.
