# Supplementary material for: Direct and Indirect Factors Influencing Cat Outcomes at an Animal Shelter
Source: Front Vet Sci. 2022 Jun 7;9:766312. doi: 10.3389/fvets.2022.766312 (PMC9211776; doi:10.3389/fvets.2022.766312)
Supplement: Supplementary file 1 [file Data_Sheet_1.PDF]

Supplementary Material

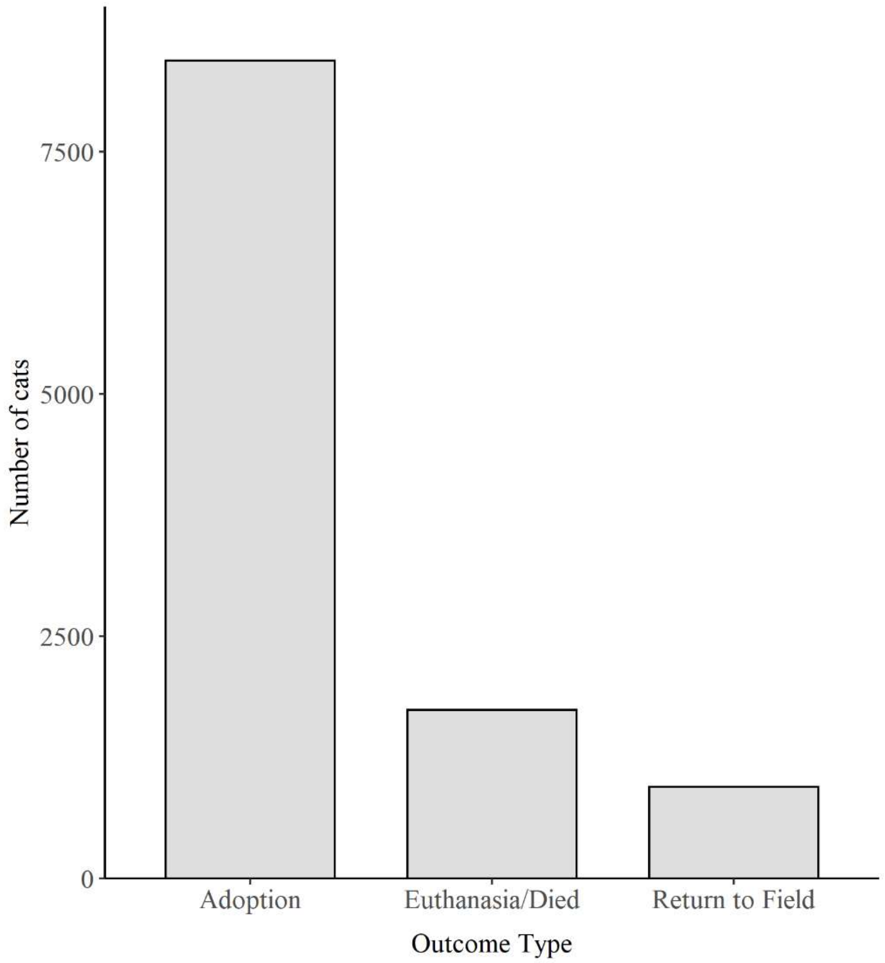

Supplementary Figure S1. Summary of outcome types.

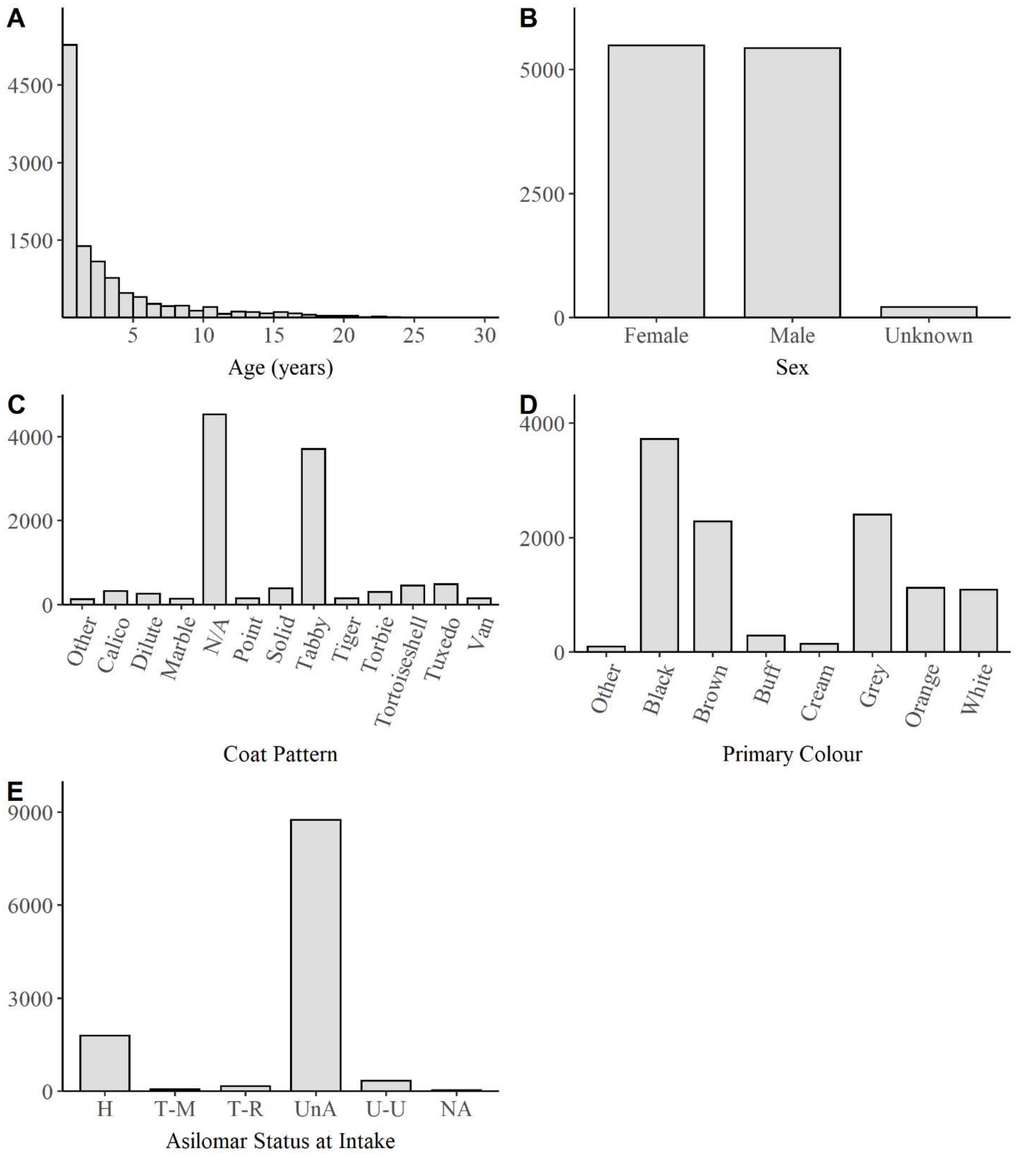

Supplementary Figure S2. Summary of physical attribute variables. Values on y-axis for all panels refer to counts of cats included in data. In panel E, H=healthy; T-M=treatable-manageable; T-R=treatable-rehabilitatable; UnA=Unassigned; U-U = unhealthy-untreatable; NA=no entry. See main text for details.

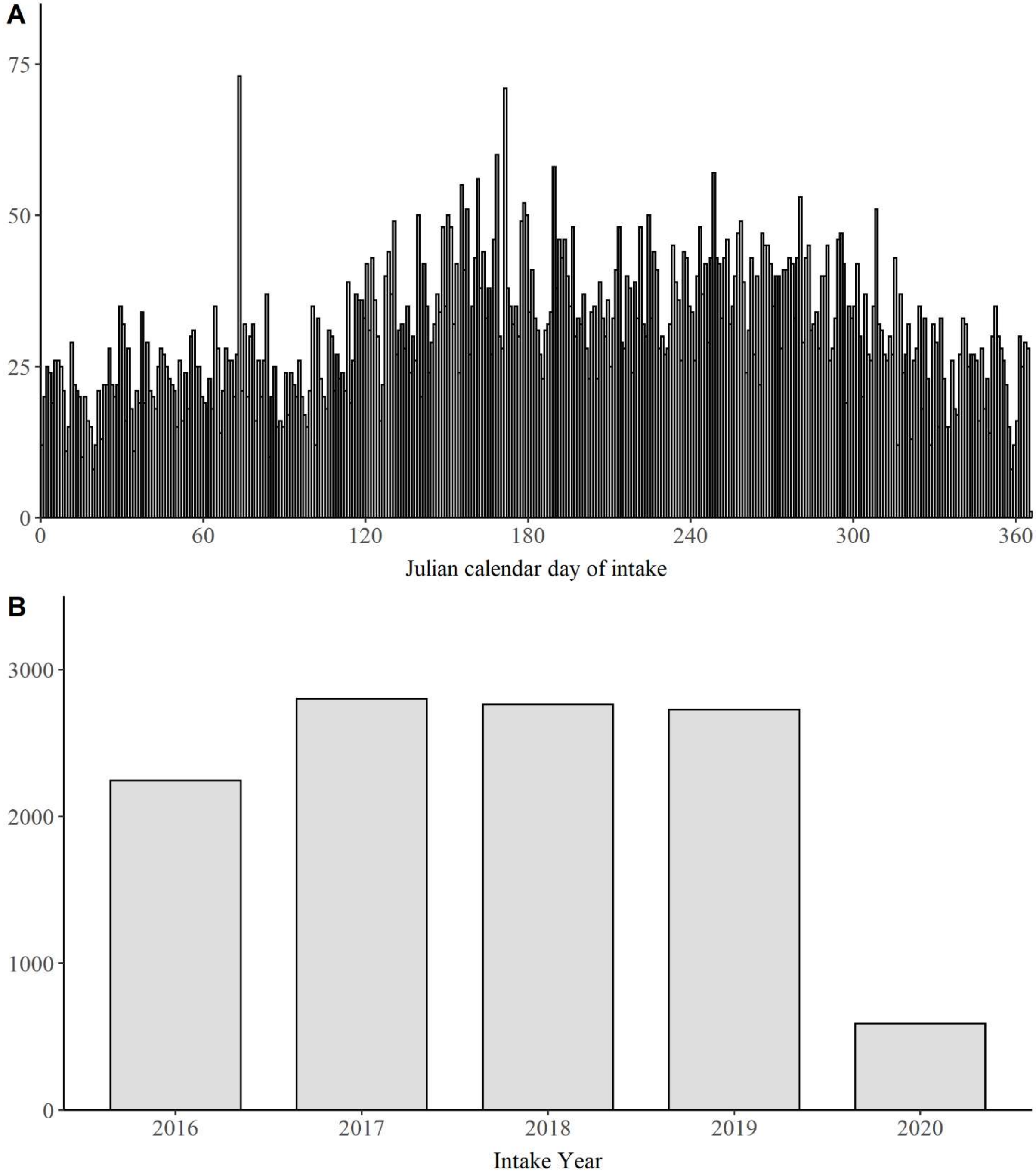

Supplementary Figure S3. Summary of intake date and year. The top panel (A) indicates the number of intakes across the Julian date, merged across years, where 1 refers to January 1 and 365 refers to December 31. The lower panel (B) describes the number of intakes across the 4 years of our study. Twelve months of data were included for 2017, 2018, and 2019, 6 months of data were incorporated in 2016 and 5 months in 2020. Low intake numbers in 2020 were related to the SARS-CoV-2 pandemic. Values on y-axis for both panels refer to counts of cats included in data.

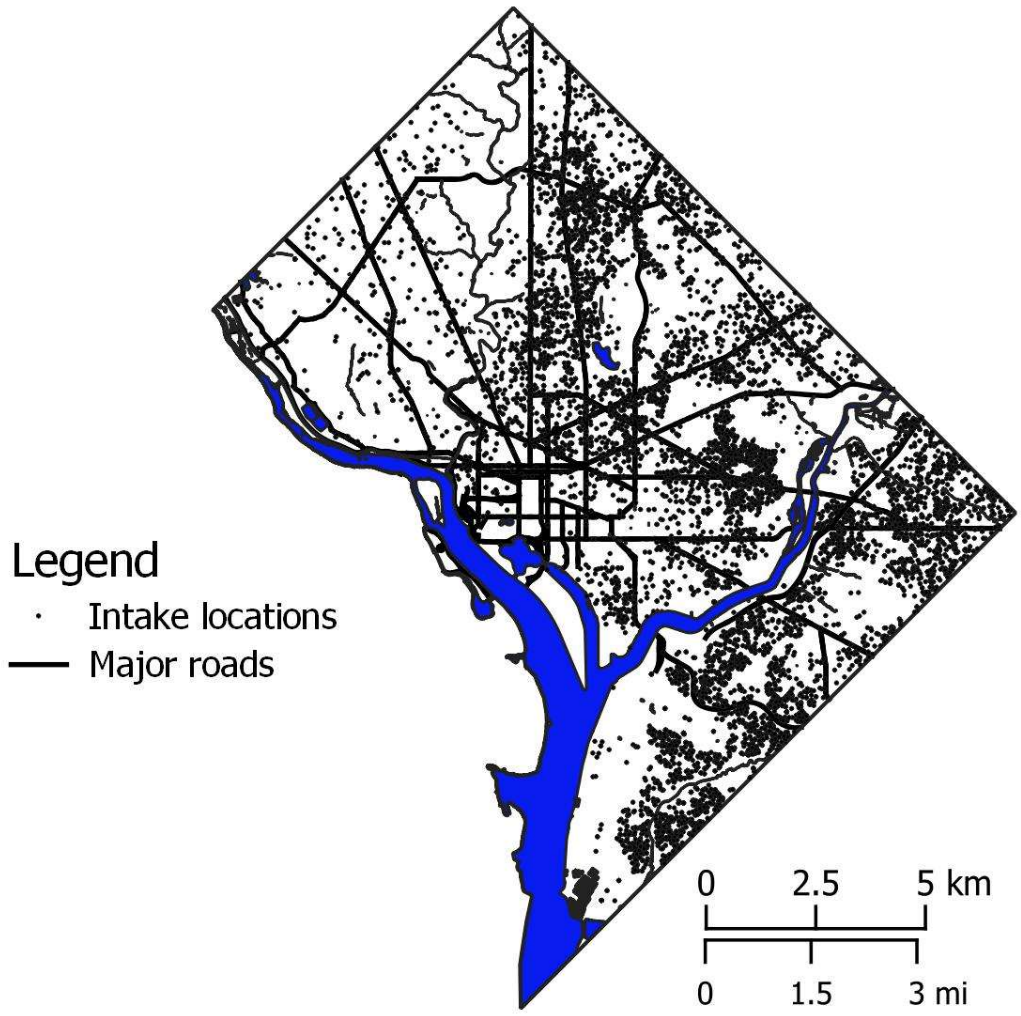

Supplementary Figure S4. A map of Washington, D.C., indicating the location of origin for cats included in our analysis.

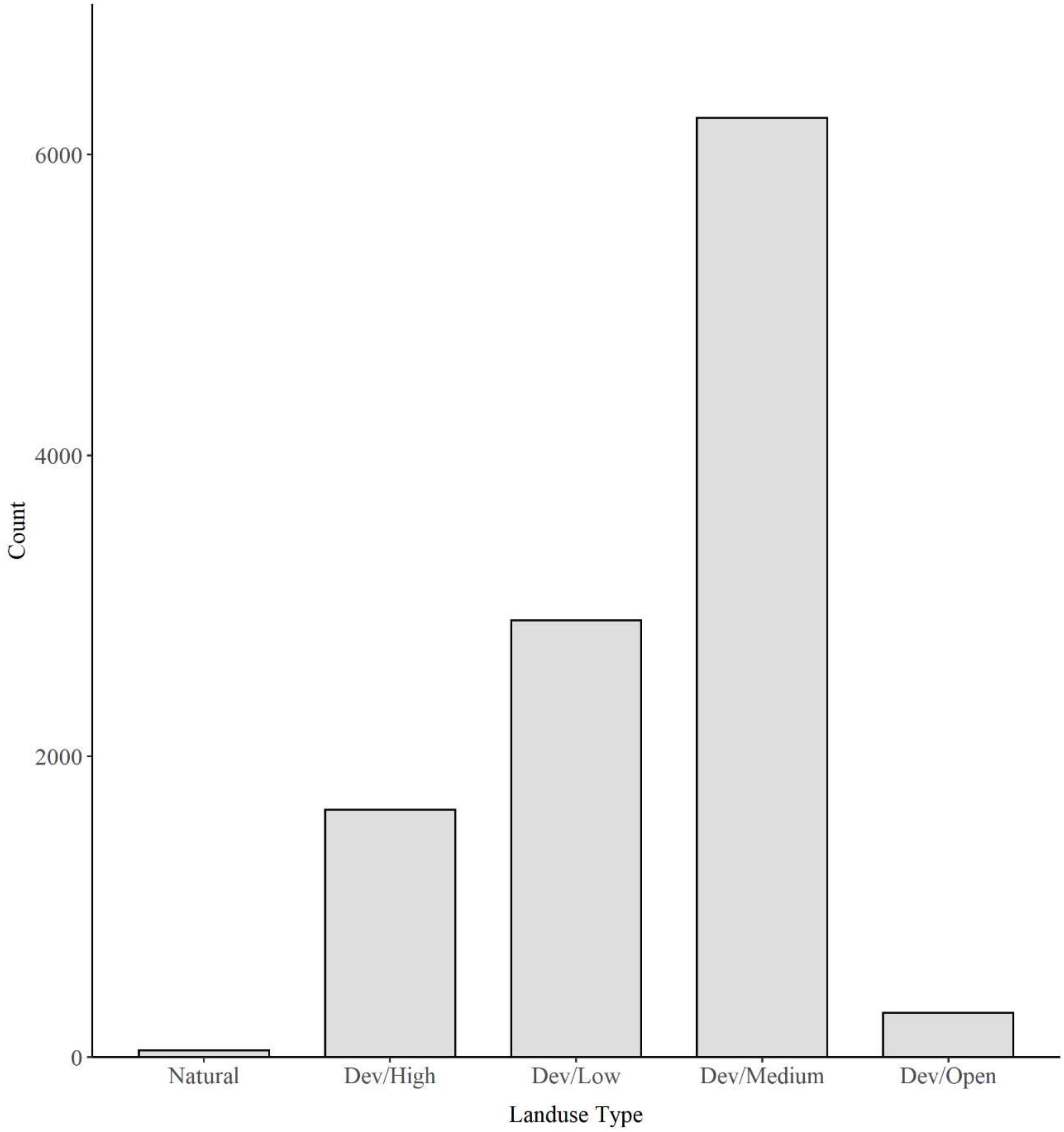

Supplementary Figure S5. A summary of landuse types. Dev/High = Developed/High Intensity; Dev/Low = Developed/Low intensity; Dev/Medium = Developed/Medium intensity; Dev/Open = Developed/Open space

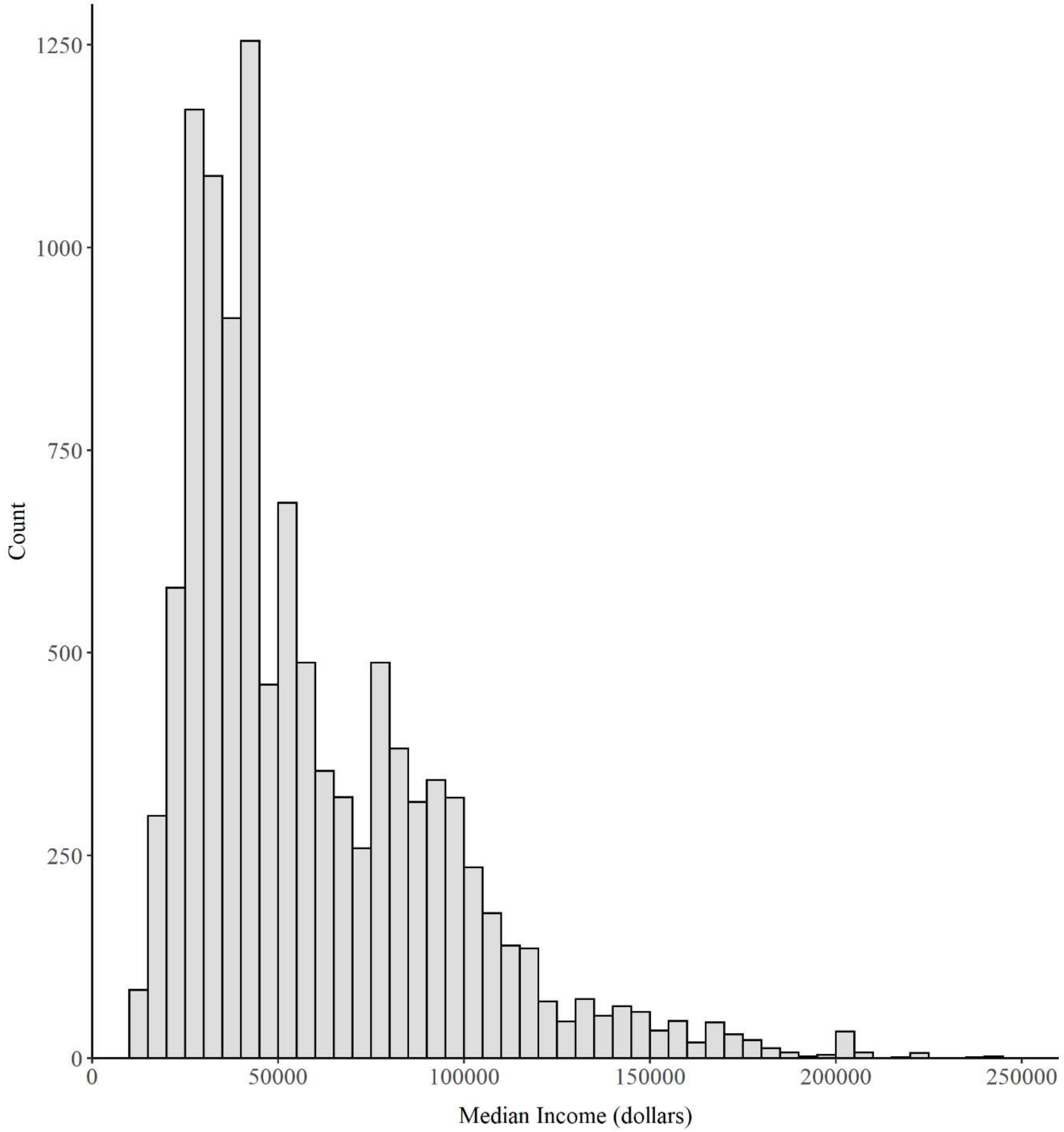

Supplementary Figure S6. A summary of median income. Each bar represents $5,000.

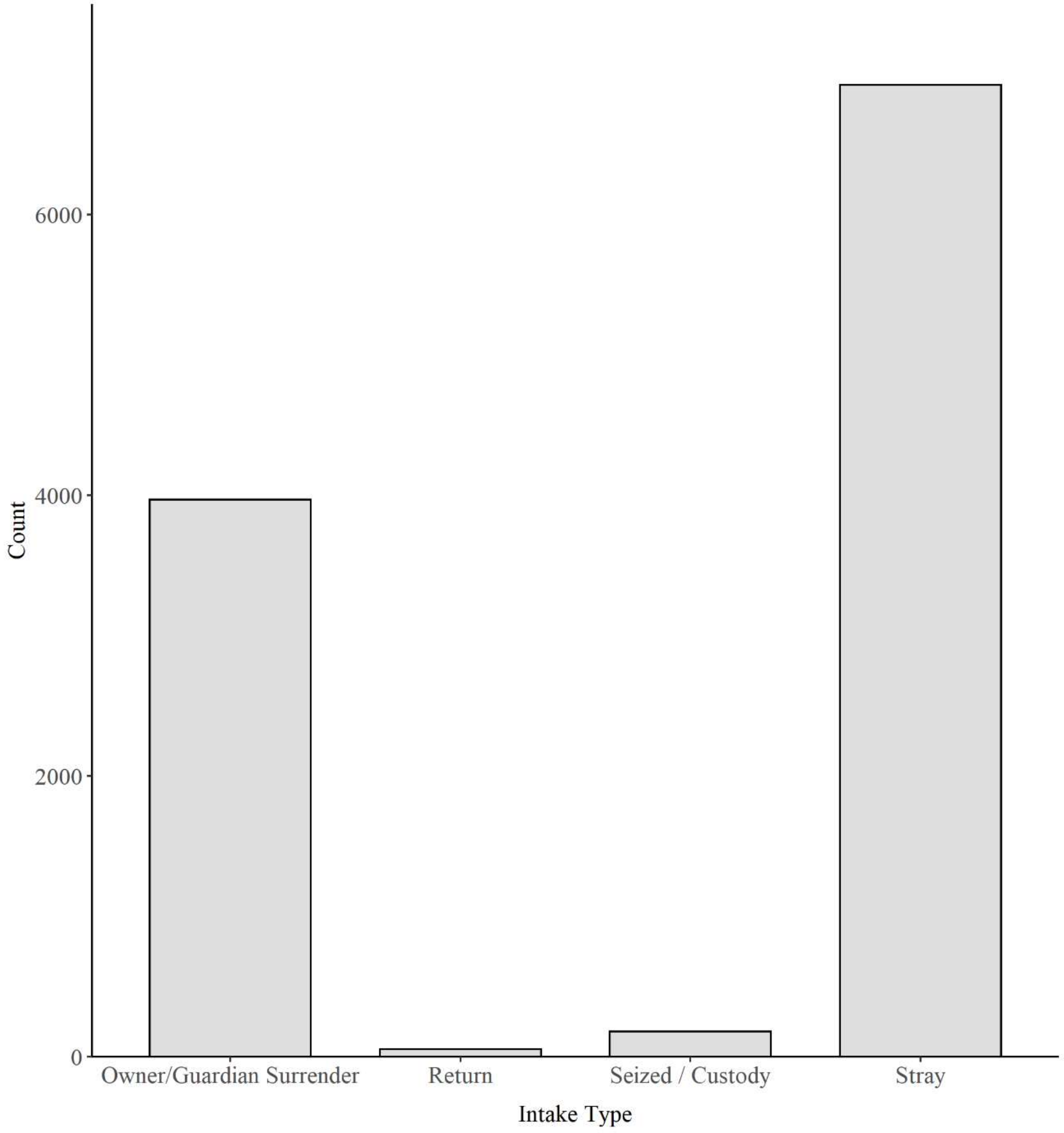

Supplementary Figure S7. A summary of the counts of intake types included in our study.

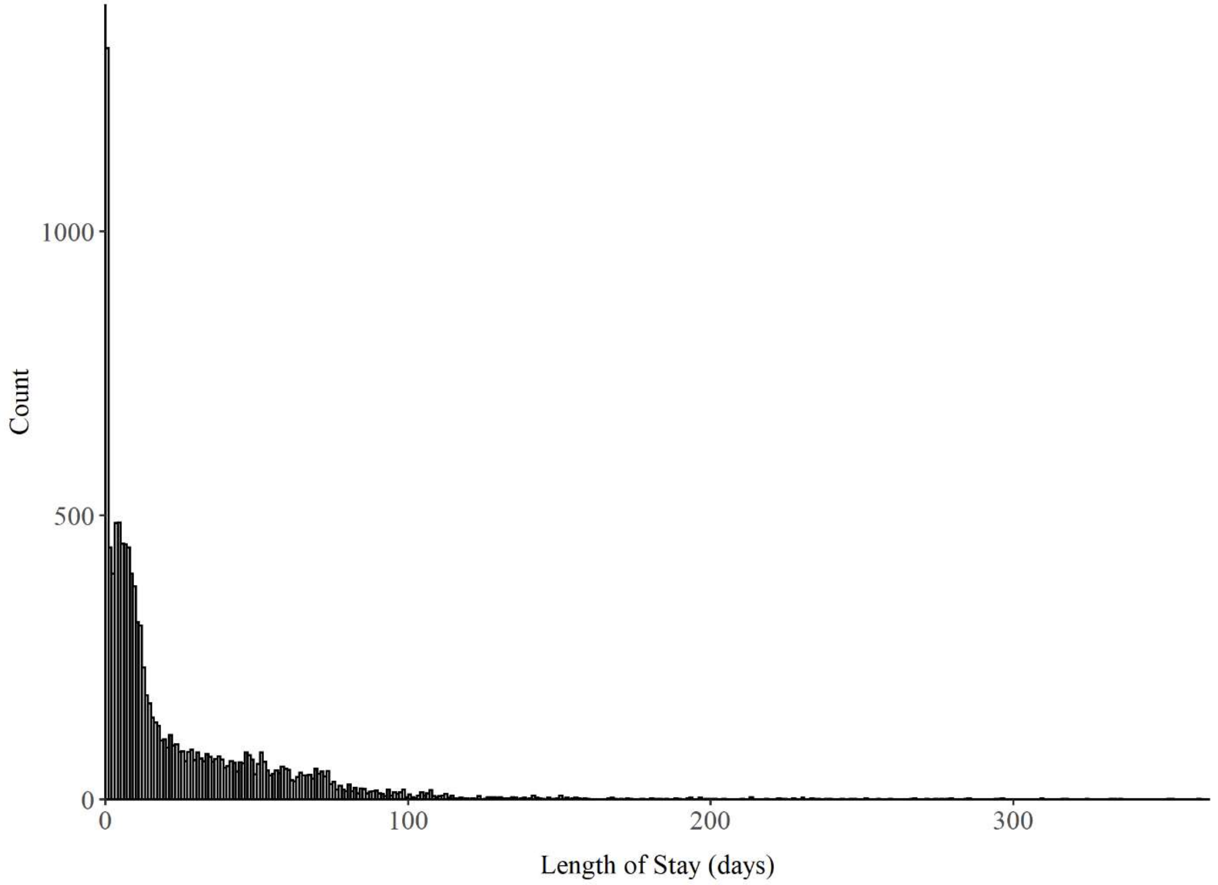

Supplementary Figure S8. Histogram depicting the length of stay in days at the Humane Rescue Alliance for cats included in our study under one year. Four cats had lengths of stay longer than one year, at 411, 439, 733 and 861 days, not depicted here. However, those individuals were included in the analyses.

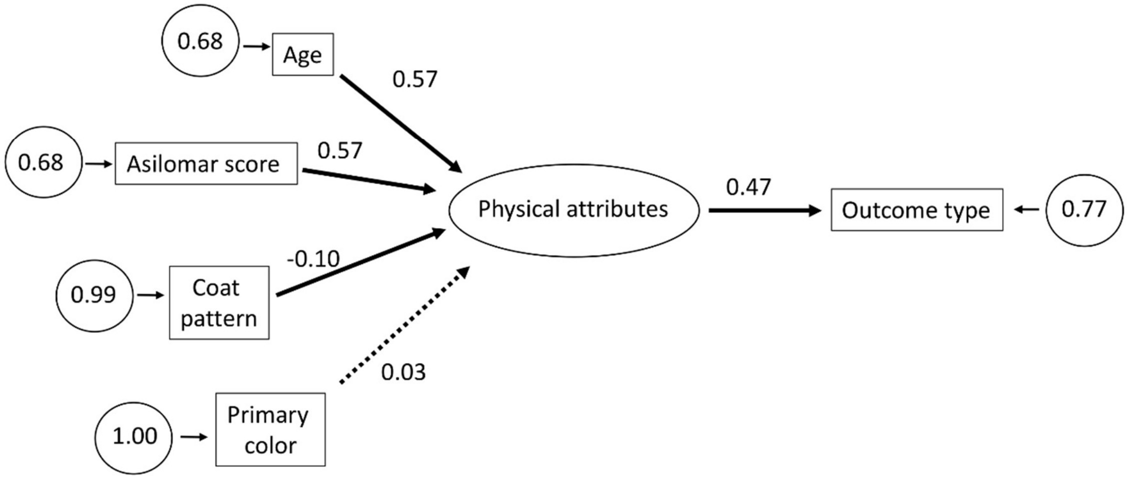

Supplementary Figure S9. The structural equation model describing Hypothesis 1 Outcome depends on the physical attributes and health status of the cat. Arrows describe the direction of effect. Solid black arrows are statistically significant at $\alpha = 0.05$, dotted arrows indicate a lack of statistical significance. Numbers alongside the arrows are standardized path coefficients (beta coefficients). Measured variables are depicted in rectangles, latent variables are depicted in ovals, error terms are in circles. Based on how they were coded, negative coefficients indicate an increased likelihood of adoption and positive coefficients indicate a decrease in likelihood of adoption.

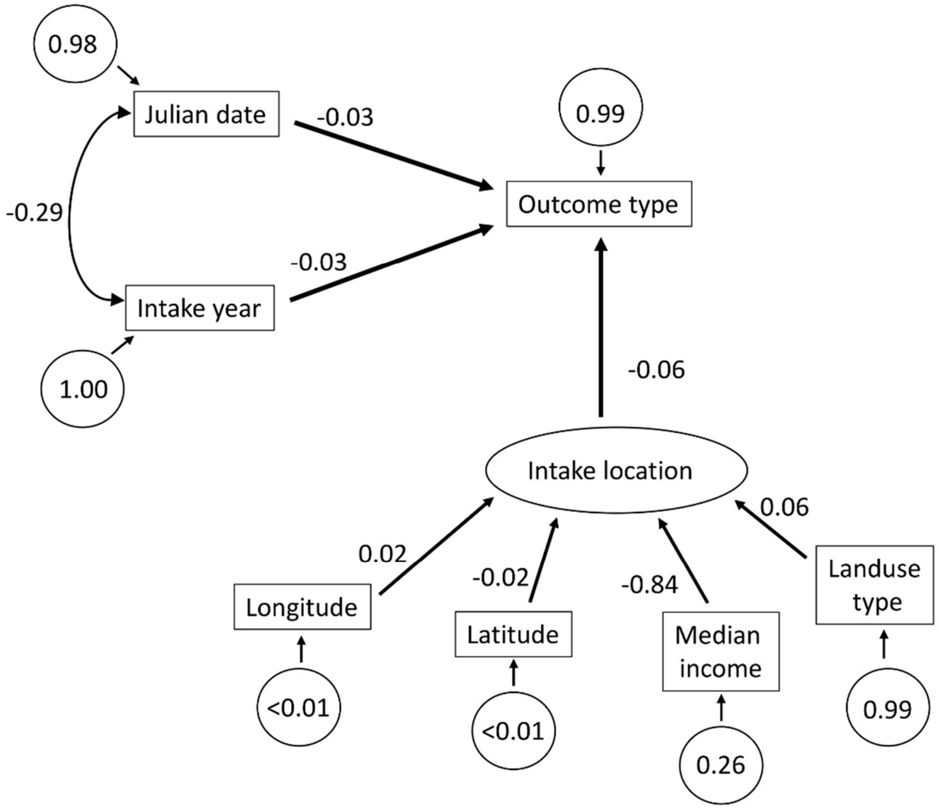

Supplementary Figure S10. The structural equation model describing Hypothesis 2 Outcome depends on the location and date of intake of the cat. Arrows describe the direction of effect. Solid black arrows are statistically significant at $\alpha = 0.05$. Numbers alongside the arrows are standardized path coefficients (beta coefficients). Measured variables are depicted in rectangles, latent variables are depicted in ovals, error terms are in circles. Based on how they were coded, negative coefficients indicate an increased likelihood of adoption and positive coefficients indicate a decrease in likelihood of adoption.

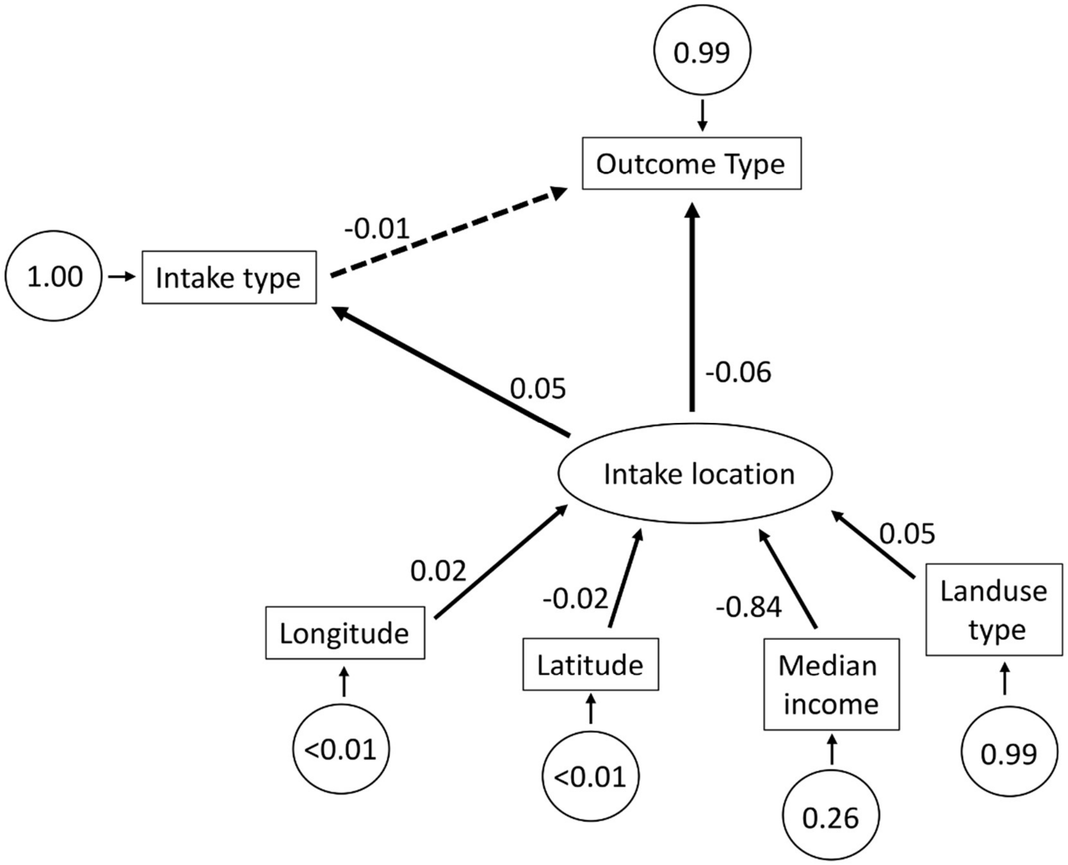

Supplementary Figure S11. The structural equation model describing Hypothesis 3: Outcome depends on human influences prior to intake, represented by intake location and intake type. Arrows describe the direction of effect. Solid black arrows are statistically significant at $\alpha = 0.05$, dotted arrows indicate a lack of statistical significance. Numbers alongside the arrows are standardized path coefficients (beta coefficients). Measured variables are depicted in rectangles, latent variables are depicted in ovals, error terms are in circles. Based on how they were coded, negative coefficients indicate an increased likelihood of adoption and positive coefficients indicate a decrease in likelihood of adoption.

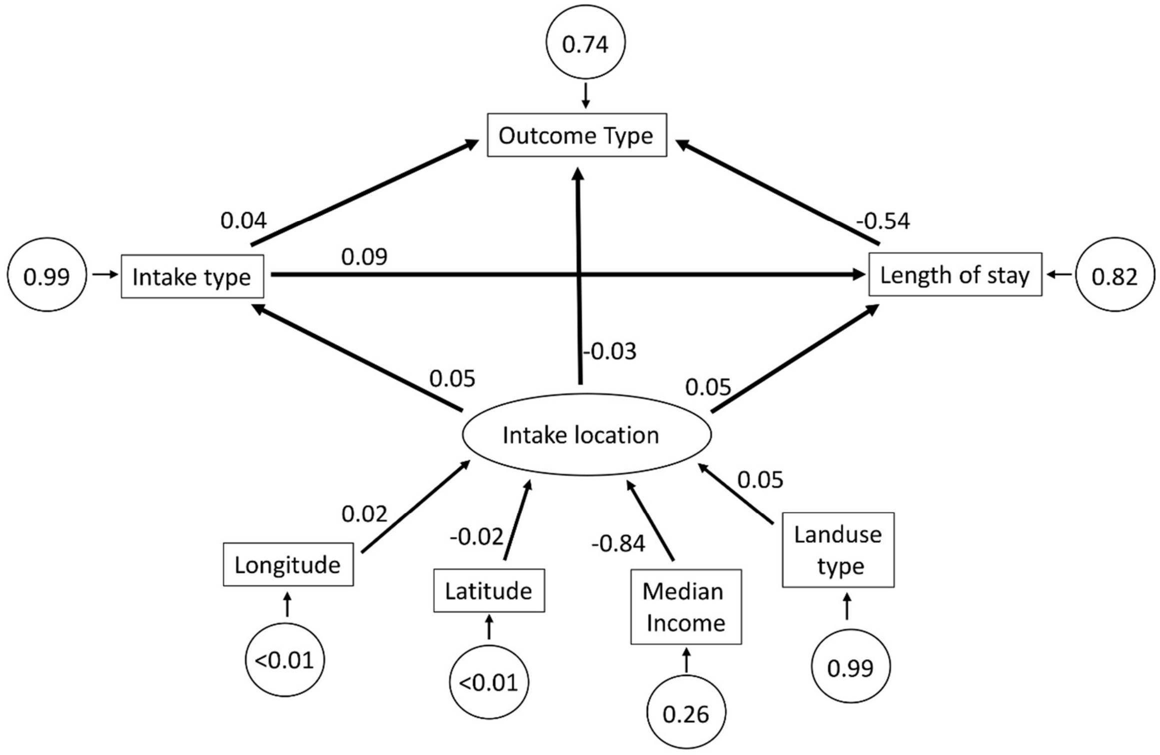

Supplementary Figure S12. The structural equation model describing Hypothesis 4: Outcome depends on experiences prior to outcome. Arrows describe the direction of effect. Solid black arrows are statistically significant at $\alpha = 0.05$. Numbers alongside the arrows are standardized path coefficients (beta coefficients). Measured variables are depicted in rectangles, latent variables are depicted in ovals, error terms are in circles. Based on how they were coded, negative coefficients indicate an increased likelihood of adoption and positive coefficients indicate a decrease in likelihood of adoption.
